# Capturing Hammerhead Ribozyme Structures in Action by Modulating General Base Catalysis

**DOI:** 10.1371/journal.pbio.0060234

**Published:** 2008-09-30

**Authors:** Young-In Chi, Monika Martick, Monica Lares, Rosalind Kim, William G Scott, Sung-Hou Kim

**Affiliations:** 1 Center for Structural Biology, Department of Molecular and Cellular Biochemistry, University of Kentucky, Lexington, Kentucky, United States of America; 2 Center for the Molecular Biology of RNA, Sinsheimer Laboratory, University of California at Santa Cruz, Santa Cruz, California, United States of America; 3 Department of Chemistry, University of California, Berkeley, Berkeley, California, United States of America; The Scripps Research Institute, United States of America

## Abstract

We have obtained precatalytic (enzyme–substrate complex) and postcatalytic (enzyme–product complex) crystal structures of an active full-length hammerhead RNA that cleaves in the crystal. Using the natural satellite tobacco ringspot virus hammerhead RNA sequence, the self-cleavage reaction was modulated by substituting the general base of the ribozyme, G12, with A12, a purine variant with a much lower pK_a_ that does not significantly perturb the ribozyme's atomic structure. The active, but slowly cleaving, ribozyme thus permitted isolation of enzyme–substrate and enzyme–product complexes without modifying the nucleophile or leaving group of the cleavage reaction, nor any other aspect of the substrate. The predissociation enzyme-product complex structure reveals RNA and metal ion interactions potentially relevant to transition-state stabilization that are absent in precatalytic structures.

## Author Summary

Enzymes use variations of a few standard approaches to catalyze reactions. One of these approaches, acid–base catalysis, is of such fundamental importance that it is common to both protein enzymes and RNA-based enzymes, or ribozymes. The hammerhead ribozyme is one such ribozyme that uses an invariant guanine residue as a general base in its catalytic reaction. By changing this to an adenine, we can slow the reaction rate 100,000-fold, permitting us to capture both active, precatalytic, and postcatalytic forms of the ribozyme. We have exploited this approach to obtain near-atomic–resolution three-dimensional structures of the hammerhead ribozyme both before and after catalytic self-cleavage. These structures provide complementary views of the chemical step of hammerhead ribozyme catalysis.

## Abstract 3

The hammerhead ribozyme exploits acid/base catalysis as an enzymatic mechanism. Weakening the general base, G12, by mutating it into an A, greatly slows the reaction. As a result, the before and after cleavage structures could be captured in a crystal lattice.

## Introduction

The hammerhead ribozyme, since its discovery in satellite virus RNA genomes [1,2], has been a central focus of experiments designed to correlate RNA structure with RNA catalysis, as it is a comparatively small RNA whose biochemistry has been intensively investigated using a wide variety of approaches [3–5]. Recently, the discovery that natural hammerhead RNAs having tertiary contacts distant from the active site may enhance catalysis up to approximately 1,000-fold relative to “minimal” hammerheads [6–9] compelled renewed mechanistic and structural investigations.

**Figure 1:**
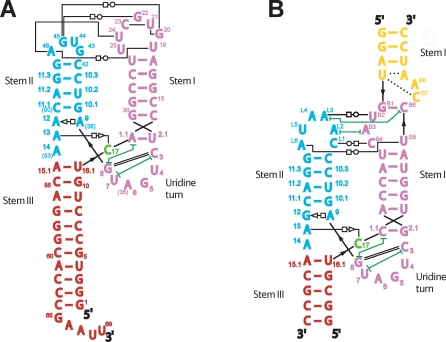
Two Classes of Full-Length Hammerhead Ribozymes Secondary and schematic tertiary structural representations of the sTRSV hammerhead (A) and the *Schistosoma* hammerhead [12] (B), depicting the two classes [6] of hammerhead ribozyme tertiary contacts.

Natural hammerhead ribozymes fall into two distinct classes [6] based upon the nature of the tertiary contacts between Stem I and Stem II (Figure 1). The most well-characterized member of the first class of natural hammerheads occurs within the satellite RNA of the tobacco ringspot virus (sTRSV), which is also the first hammerhead ribozyme discovered [10]. The best-characterized member of the second class of natural hammerheads occurs within the multimeric RNA transcript of the Schistosoma mansoni alpha repetitive sequence (Smα) repetitive DNA within the S. mansoni genome [11,12]. The structure [13] of a full-length Schistosome hammerhead [12] ribozyme-competitive inhibitor complex in which a substrate analog having a modified 2′-OMeC17 nucleophile was recently obtained, revealing how G12 becomes positioned to initiate cleavage as a general base, and how G8 may function as a general acid in hammerhead ribozyme catalysis. However, the substrate was inactivated by replacing the nucleophilic 2′-OH of the cleavage-site nucleotide (C17) with an inert ether linkage, thus potentially altering the active site environment.

**Figure 2:**
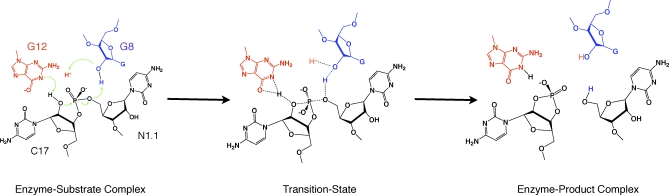
The Hammerhead Ribozyme Self-Cleavage Reaction Schematic diagram of the enzyme–substrate, transition-state, and enzyme–product complexes of an unmodified hammerhead active site, interpolated from the 2GOZ structure in which G12 (red) is positioned to function as a general base in the cleavage reaction, and G8 (blue) is positioned consistent with a possible role in acid catalysis. To function as a general base, the N1 of G12 must be deprotonated (as shown), and it can then abstract the 2′-H from C17 (in black) to generate the nucleophile. The 2′-OH of G8 (in blue) is positioned to donate a proton to the 5′-O of residue N1.1, the leaving-group in the self-cleavage reaction. Green arrows represent electron pairs that mediate proton transfer and covalent bond breakage and formation. The transition state consists of a trigonal bipyramidal oxyphosphorane in which the nucleophile and leaving group occupy the axial positions. Partial bond formation and breakage is indicated with dotted lines. The products of the cleavage reaction possess 2′,3′-cyclic phosphate and 5′-OH termini as shown. The 2′,3′-cyclic phosphate is not hydrolyzed by the ribozyme, and in the structure, it is found in the form of a predissociation complex. In the sTRSV hammerhead structure, the G12A modification results in a much weaker base, but one that is not protonated at N1. The nucleotide N1.1 is not conserved. In 2GOZ, it is C1.1, and in the sTRSV hammerhead, it is an A.

We have now obtained two crystal structures from a full-length sTRSV hammerhead RNA with an unmodified cleavage site that has an active nucleophile. These include an active enzyme–substrate complex trapped just prior to catalytic cleavage from freshly grown crystals, and an active enzyme–product complex trapped prior to dissociation of the product, subsequent to cleavage (Figure 2) from crystals allowed to age for several weeks.

Instead of inactivating the nucleophile via methylation, as was done with the Schistosome hammerhead [12], the cleavage reaction in the case of the sTRSV hammerhead has been greatly decelerated with a G12A enzyme active site variant that lowers the pK_a_ of the purine general base in the cleavage reaction from approximately 9.5 to approximately 3.5, which, assuming the observed log-linear rate dependence [6,14,15] on pH, potentially represents an approximately 10^6^-fold decrease of the reaction rate. The G12A mutation in the context of a minimal hammerhead ribozyme has been reported previously to create a greater than 500-fold reduction in the cleavage rate [16]. More recently, a full-length peach latent mosaic viroid hammerhead ribozyme with a G12A substitution has been shown to have very limited cleavage activity [17]. We have measured an approximate 10^−6^-fold rate reduction for the G12A substitution in the full-length hammerhead, and have also shown the G12A modification retains the standard pH dependence of the hammerhead reaction rate (cf: Figure S4). The correlation between the pK_a_s of various purine derivatives substituted at position 12 and the hammerhead ribozyme cleavage rate has been thoroughly examined using inosine, diaminopurine, and 2-aminopurine nucleotides substituted for G12 [18]. These results are all consistent with the purine at G12 functioning as a general base, as well as with the G12A mutant being a very poor, but not completely inactive, general base. By greatly slowing the reaction, the hammerhead RNA crystallizes prior to cleavage, but remains active in the crystal and slowly cleaves. We have exploited this property to obtain both reactant (precatalytic) and product (postcatalytic) structures of the active hammerhead ribozyme to 2.4 Å and 2.2 Å resolution, respectively.

## Results and Discussion

In our study, two datasets were used; one, the reactant, diffracts to 2.4 Å resolution and the other, the cleavage product, diffracts to 2.2 Å resolution. In both datasets, two crystallographically independent 69-nucleotide hammerhead structures (Figure S1) occupy a P1 unit cell (a = 27.9 Å, b = 53.0 Å, c = 72.0 Å, α = 74.6°, β = 81.4°, γ = 75.6°) [19]. The only significant difference between molecule 1 and molecule 2 within the asymmetric unit is in the tertiary contact region, where the electron density for several of the nucleotides involved in the tertiary contact in molecule 2 is quite weak, indicating disorder and dynamic flexibility in a structure otherwise characterized by a well-resolved and easily interpretable electron density map. Two precatalytic (uncleaved) models were unambiguously constructed in the 2.4 Å electron density map and refined. Refinement of the reactant structure of the 2.4 Å data (Tables 1 and 2) clearly shows that both molecules in the asymmetric unit are in an uncleaved, precatalytic state, whereas both molecules in the asymmetric unit of the product 2.2 Å structure (Tables 1 and 2) are in a cleaved, postcatalytic state.

**Table 1:**
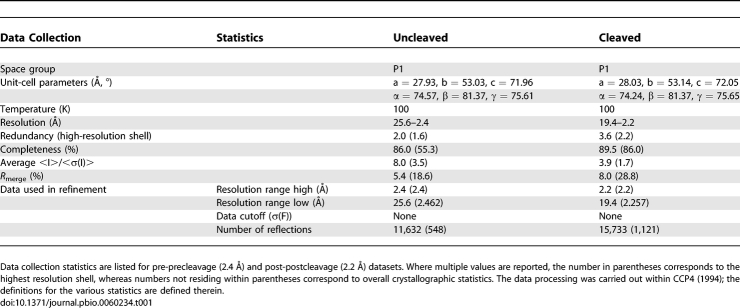
Data Collection Statistics

**Table 2:**
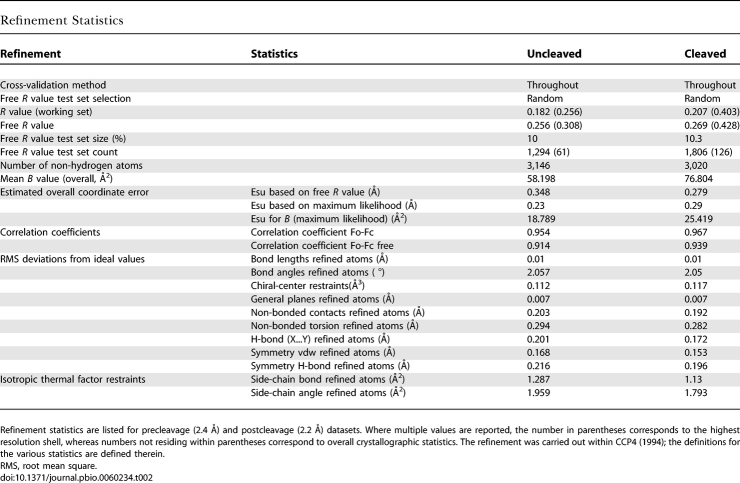
Refinement Statistics

### The Hammerhead Enzyme–Substrate Complex Structure

**Figure 3:**
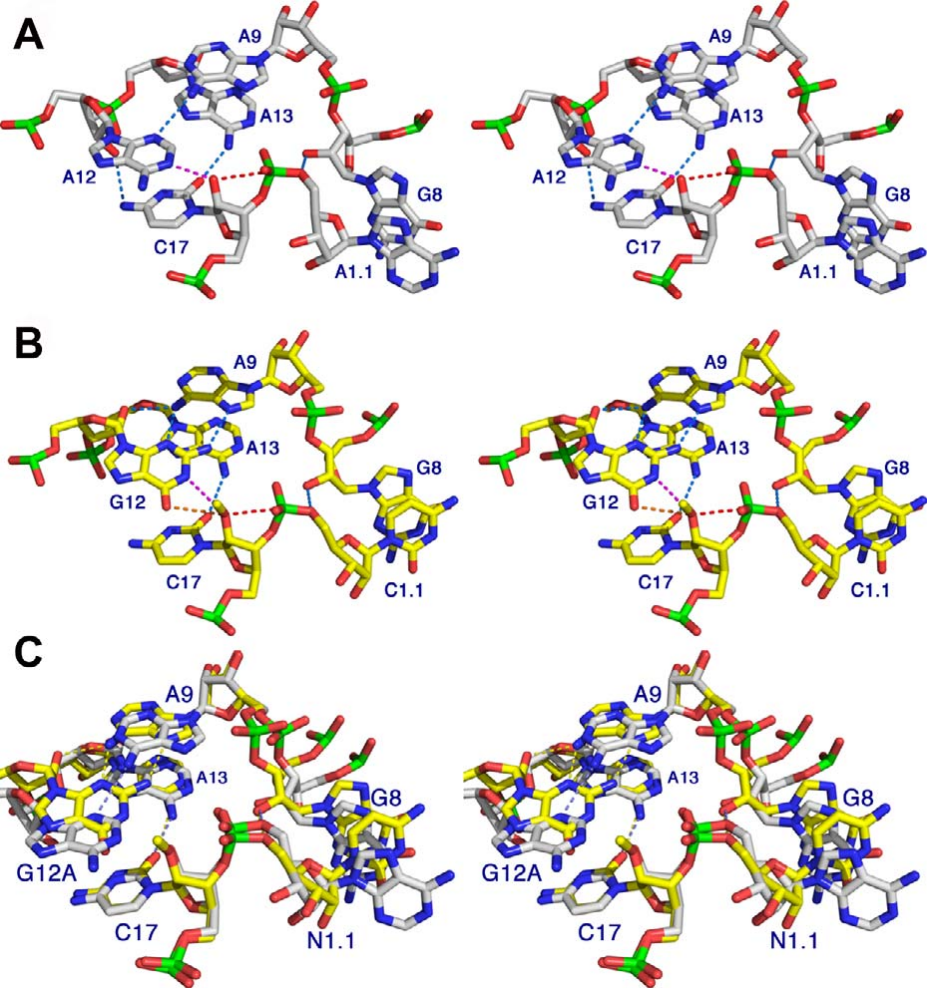
The Hammerhead Ribozyme Reactant and Product Active Sites (A and B) Stereo views of two hammerhead ribozyme active sites [37]. The active site of the uncleaved G12A sTRSV hammerhead (A) with an unmodified nucleophile, and the Schistosome hammerhead 2GOZ [12] (B) with a 2′-OMe modification of the nucleophilic 2′-oxygen of C17. Hydrogen bonds are shown as light-blue dotted lines, the trajectory of bond formation is indicated as a red dotted line, and potential “active” hydrogen bonds in base catalysis are indicated as pink and orange dotted lines. (C) depicts a superposition of (A) and (B).

The precleavage or enzyme–substrate complex structure of the G12A sTRSV hammerhead RNA at 2.4 Å resolution reveals an active site (Figure 3A) very similar to that of the Smα hammerhead (Figure 3B), despite the presence of the 2′-OMe modification in the latter, and the G12A substitution in sTRSV hammerhead. Hence, it is reasonable to conclude that neither modification grossly perturbs the atomic structure of the hammerhead ribozyme active site. In this sense, the uncleaved sTRSV hammerhead and the Smα hammerhead structures are both useful internal experimental controls that put to rest any concerns that either the previous 2′-OMe modification or the current G12A substitution induces formation of a catalytically incompetent hammerhead ribozyme structure. A thorough analysis of two decades of experimental results obtained from biochemical and mechanistic investigations of the hammerhead ribozyme has been carried out [20,21] that confirms the assessment that the Smα hammerhead active site conformation, and therefore the similar sTRSV hammerhead active site conformation, indeed represent the catalytically competent structural state.

Some small differences between the sTRSV hammerhead enzyme–substrate complex structure and the corresponding Smα hammerhead enzyme–inhibitor complex do exist (Figure 3C). The unmodified 2′-OH of C17 in the latter appears to be slightly more in-line with the scissile phosphate (168.5° vs. 162°), and the position of A12 differs slightly, due to a different hydrogen-bonding interaction with A9 that replaces the G12/A9 sheared pairing (Figure 3A–3C). The primary difference is that the hydrogen bond between the exocyclic amine of G12 and N7 of A9 is, by necessity, absent in the G12A structure, so that only one hydrogen bond between A9 and A12 exists (Figure 3A) rather than three (Figure 3B). The net effect is that the positions of A9 and A12 in the G12A sTRSV structure change slightly compared with the G12 structure (2GOZ), as can be seen in the superposition of the active site residues (Figure 3C). The difference in absolute positions of the scissile phosphorus in the two superimposed structures is 1.7 Å. The geometry of the G12A sTRSV appears to be somewhat better suited to initiation of the cleavage reaction. Specifically, the angle between the N1 of A12, the 2′O nucleophile (C17), and the adjacent scissile phosphorus is 149°, and the distance between N1 and O2′ is 2.7 Å. The corresponding angle in the G12 structure with the modified substrate (2GOZ) is 139° and the N1 to O2′ distance is 3.5 Å. The in-line attack angle (between O2′, P, and O5′) is 168° in the G12A structure, versus 162° in the previous G12 structure. Hence, the slow cleavage rate appears to be primarily due to the result of the purine pK_a_ shift from approximately 9.5 to approximately 3.5 upon G12A substitution, rather than due to a disadvantageous structural perturbation. Deprotonation of G12 must occur (Figure 2) to initiate the cleavage reaction, but G12 is almost certainly protonated in the 2GOZ crystal structure at pH 6.5, whereas A12 is normally deprotonated at neutral pH. In this sense, A12 may be a better (albeit much slower) representation of the activated ribozyme poised for general base catalysis, even though A12 is a much weaker base than G12 due to its much smaller pK_a_.

### The Hammerhead Enzyme–Product Complex Structure

**Figure 4:**
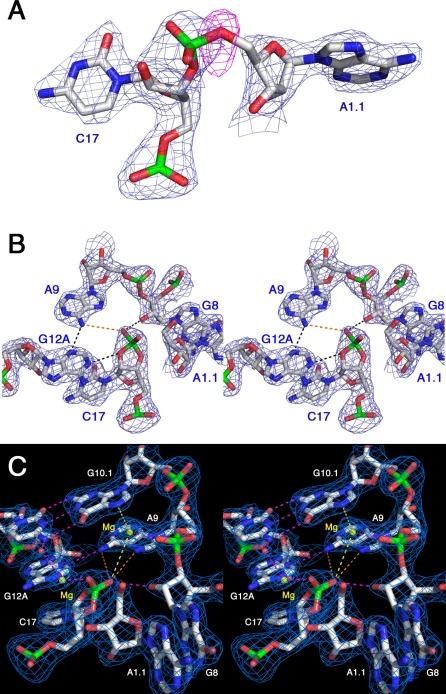
Hammerhead Ribozyme Cleavage in the Crystal (A) Refining the uncleaved structure against the cleavage-product data produces a negative residual (or *F*calc − *F*obs) difference peak (shown in red, contoured at 3 σ) centered on the 5′-oxygen, the leaving group of the cleavage reaction. A gap in the 2Fo-Fc map (shown in blue, contoured at 1.0 σ) is apparent, despite model bias from the uncleaved structure. This appears in both crystallographically independent molecules in the asymmetric unit. (B) The refined cleaved structure makes a better fit to the electron density. (C) A stereo view of the active site of the hammerhead ribozyme, showing potential (yellow and orange dotted lines) and actual (pink dotted lines) bonding interactions involving two Mg^2+^ ions (yellow spheres) and the RNA. The potential interactions may form stabilizing contacts when the scissile phosphate is in the trigonal bipyramidal oxyphosphorate transition state, helping to dissipate excess negative charge. In particular, the invariant A9 may engage in transition-state stabilization interactions in extrapolation from the product structure, as indicated by the orange dotted lines.

Refinement of a hypothetically uncleaved structure using the 2.2 Å resolution cleavage product dataset, obtained from the crystals allowed to age, revealed unique and significant (>3 σ) negative difference Fourier peaks (Figure 4A) positioned directly on the O5′ atoms of A1.1 of each molecule of the hypothetically uncleaved model (without noncrystallographic symmetry averaging applied), in addition to clear breaks in the sigma-A–weighted 2Fo-Fc maps [22–25] at the same locations (Figures 4B and S1B), thus demonstrating that the substrate RNA is predominantly in the cleaved state. The negative difference Fourier peak on molecule 1 (Figure 4A) is slightly more pronounced, and subsequent refinement of the structure in which a 2′,3′-cyclic phosphate was added to C17, and the phosphate linking it to A-1.1 was replaced with a terminal 5′-OH, provided a much better fit to the observed electron density (Figure 4B and 4C). Molecule 1 appears to be completely cleaved, whereas a small amount of molecule 2 may remain in the uncleaved form. Cleavage of molecule 2 is thus best interpreted as somewhat incomplete, and it is notable that possibly less-complete cleavage corresponds to the molecule in which the tertiary contact is less well defined, hinting that the tertiary contact may function as a molecular modulator in the life cycle of the satellite virus RNA that regulates cleavage and possibly religation activities. The internal equilibrium of the sTRSV hammerhead ribozyme greatly favors the cleaved over the uncleaved state, whereas the internal equilibrium of the Smα hammerhead is such that about 1/3 of the RNA is ligated [26].

The cleaved structure reveals several interactions potentially relevant to the catalytic mechanism (Figure 4C). In molecule 1 of the cleavage product structure, two Mg^2+^ ions appear to interact with the scissile phosphate, which is in the 2′,3′-cyclic form. In addition, the 2′-OH of G8, previously implicated as possibly the acid catalyst [13,18], makes a hydrogen bond to the more proximal nonbridging phosphate oxygen of the cyclic phosphate. N1 and N6 of A9 are also positioned about 4.5 Å from the same nonbridging cyclic phosphate oxygen atom, as is the Mg^2+^ ion bound to A9 phosphate. Although these latter distances, shown as orange and yellow dotted lines in Figure 4C, are too large to form bonding interactions in the product structure, it is plausible that they form stabilizing interactions within the trigonal bipyramidal oxyphosphorane transition-state structure to help disperse transiently accumulating excess negative charge, thus contributing to catalysis. (An analogous role for adenosine bases is observed in the hairpin ribozyme [27], and a requirement for either divalent metal ions or a high concentration of positive charge [28] in the hammerhead cleavage reaction is well known.) A second Mg^2+^ ion is observed in molecule 1 to coordinate directly with the other nonbridging cyclic phosphate oxygen, suggesting a possible role for the second Mg^2+^ ion in stabilizing the cleavage product or transition state. Although a single divalent metal ion has yet to be observed in a hammerhead crystal structure to bridge the scissile and A9 phosphates via a predicted inner-sphere coordination [29], the observed Mg^2+^ ion and A9 nucleotide base interactions nonetheless suggest how transition-state stabilization, especially at low ionic strength, may be facilitated. Since this postcatalytic structure represents the state of the molecule before product dissociation, due to trapping by the crystal lattice, we suggest that the structure reveals features relevant to the transition state and that are complementary to those in the uncleaved state.

### Structures of the Hammerhead Tertiary Contact

**Figure 5:**
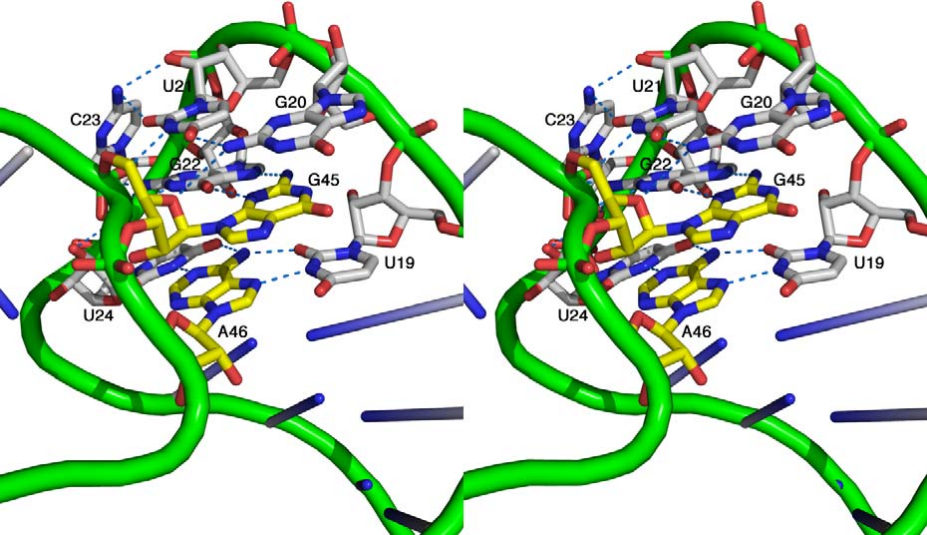
Hammerhead Ribozyme Tertiary Contacts Close-up stereo view of the tertiary interactions between Stems I and II in the sTRSV hammerhead RNA. The trace of the phosphodiester backbone is represented as green tubes, and the nucleotides that participate in tertiary contacts between Stem I and Stem II are shown explicitly as atomic color-coded stick figures. Carbon atoms in the Stem I nucleotides are white, and carbon atoms in the Stem II nucleotides are yellow. Nitrogen atoms in both cases are dark blue, oxygen atoms are red, and phosphorus atoms are green. Hydrogen bonds are shown as blue dotted lines. Figure S2A and S2B depict complementary views.

The structure of the *Schistosoma* Smα hammerhead [13] revealed how the distal tertiary contacts stabilize a conformational change (relative to the minimal structure) within the active site of the hammerhead ribozyme. However, most of the naturally occurring viral hammerhead RNAs, including the sTRSV hammerhead, belong to the other class of hammerhead ribozymes in which a tetraloop on Stem II (typically the thermodynamically favored GNRA tetraloop) interacts with a closed loop on Stem I [6]. The Smα hammerhead and the sTRSV hammerhead tertiary contacts induce what are nearly identical conformational changes in the ribozyme's catalytic core, despite the fact that the sequences and structures of the two tertiary contact regions are radically different. In fact, only one tertiary base pair is common to both classes of hammerhead tertiary contacts (Figures 5, S2, and S3).

In both classes of hammerhead tertiary contacts, an apparently conserved [6] Hoogsteen base pair forms between an A in Stem-Loop II and a U in the nonhelical region of Stem I. The A in the Hoogsteen pair corresponds to position 46 in the sTRSV hammerhead and L6 in the Smα hammerhead, and the U corresponds to position 19 in the sTRSV hammerhead and B5 in the Smα hammerhead. Of the 13 natural hammerhead sequences considered in previous modeling studies [6], *all* possess this final A in the GNRA tetraloop capping Stem II, and ten possess this U adjacent to residue 1.6, suggesting the AU Hoogsteen pair is conserved due to its functional relevance, despite the fact that it evaded identification [6] before now. (The remaining three sequences have C instead of U, which can form an analogous Hoogsteen pair if protonated.) In the new sTRSV hammerhead crystal structure, the conserved AU Hoogsteen pair is found within a base triple in which another (apparently nonconserved) U from the Stem I loop forms an additional Watson-Crick base pair with the A from the Stem II loop (Figures 5, S2, and S3).

### Concluding Remarks

Until 2003, it was not recognized that a tertiary contact region possessing little recognizable sequence conservation is critical for optimal catalysis [6,7], and subsequently, it was discovered that the tertiary contacts, which impart an approximately 1,000-fold rate enhancement, induce a dramatic conformational change within the hammerhead ribozyme active site that activates it for catalysis [13]. We report here the first to our knowledge, full-length hammerhead ribozyme crystal structures in which the crystallized molecule is catalytically active, permitting capture of both the active precleavage enzyme–substrate and the postcleavage enzyme–product complexes. The former appears to be in an active conformation immediately preceding catalysis, and the latter is in a predissociated state that immediately follows catalysis. Each, therefore, provides complementary views of the unobservable transition state, the former immediately before, and the latter immediately after formation of the transition state, providing new mechanistic insights into ribozyme catalysis.

## Materials and Methods

### RNA synthesis and crystallization.

RNA sample preparation and crystallization have been previously reported [19]. Briefly, using in vitro transcription from a synthetic DNA template derived from the sequence of the sTRSV, a self-cleaving hammerhead ribozyme (69 nucleotides long) was synthesized. Since the wild-type transcription product cleaved to completion in the transcription reaction, a sequence having a mutation at position 51 (a G12A modification, using the canonical hammerhead numbering scheme), which resulted in a greatly reduced rate of cleavage, was transcribed and crystallized. The sample was purified on a fast protein liquid chromatograph (FPLC) using a diethyl aminoethyl (DEAE) ion exchange column, and the crystals were obtained by vapor diffusion as previously described [19]. For data collection, 30% (v/v) MPD was added gradually to the mother liquor, equilibrating the crystal-containing drops stepwise over a period of 3 d, before being flash frozen by liquid nitrogen stream. The cleavage-product 2.2 Å dataset, in which the RNA is predominantly cleaved, resulted from crystals that had aged substantially longer than the crystals used to collect the reactant dataset.

### Structure determination and refinement.

The native datasets from single crystals were collected at 100 K on an R-AXISIIC imaging plate detector coupled with a Rigaku Rotaflex X-ray generator and the MSC/Yale mirror optics. The datasets were processed with DENZO [30] and scaled with rotavata/agrovata implemented in the CCP4 program suite [24,31]. The final data statistics are shown in Table 1. The approximately 10% overall incompleteness was due primarily to the absence of crystal symmetry (the space group is P1).

The reactant crystal structure was determined to 2.4 Å resolution by piecewise molecular replacement using multiple copies of a seven base-paired poly-adenine standard A-form double helix (stem) as a model. The initial crystal content analysis indicated that there are two sTRSV hammerhead molecules in the asymmetric unit (*V*_M_ = 2.23 Å^3^ Da^−1^, 56% solvent content, assuming RNA density is 1.7 g/cm^3^ [32,33]. The molecular replacement search for six stems of which three potentially constitute one hammerhead structure was carried out by Phaser [34] and a solution (*Z*-score 7.7 for a translation function that was 15% higher than the next possible solution) was obtained. Subsequent rigid body refinement using CNS [35] resulted in the *R*_free_ and *R* values of 46.1% and 49.6%, respectively. The phases were improved by rounds of manual rebuilding and composite omit map calculation implemented in CNS, which enabled building of more than 80% of the structure. Finally, the proper connections and the correct sequence registrations were made with the aid of a newly determined full-length Smα hammerhead structure [13]. The subsequent refinement was carried out by Refmac [22] and Phenix [25,36]. The model was constructed, and Mg^2+^ and water sites were identified and validated using COOT [23].

The cleavage-product structure was then solved using the coordinates of the refined, uncleaved structure. The cleaved state of the substrate was identified using sigma-A–weighted (*F*_calc_-*F*_obs_) difference Fourier maps calculated in both Refmac and Phenix, and then displayed in COOT. The detailed refinement statistics are shown in Table 2.

## Supporting Information

Figure S1 Composite Omit Electron Density Map (A) Stereo view of a composite omit electron density map of the cleaved form of the hammerhead ribozyme at 2.2 Å resolution contoured at 1.0 root mean square deviation (RMSD). Each omit fragment in the composite was generated by omission of a unique 10% of the RNA structure, followed by simulated annealing refinement of the remainder of the structure (starting temperature 4,000 K) to reduce model phase bias, within the crystallographic refinement program CNS v. 1.2. [35].
(B) shows a close-up view of the active site of molecule 1.
(6.59 MB TIF)

Figure S2 Hammerhead Ribozyme Tertiary Interactions Overall stereo view of the sTRSV hammerhead backbone structure, with the nucleotides involved in the tertiary contacts shown explicitly.
(752 KB TIF)

Figure S3 Close-Up of Hammerhead Ribozyme Tertiary Interactions Close-up stereo view of the tertiary contacts shown in Figure S2, similar to Figure 5 (but without the backbone cartoon).
(1.2 MB TIF)

Figure S4 Kinetic Analysis of the G12A Mutation in the Full-Length Hammerhead Ribozyme (A, B, and C) are the results of experiments that measure the rate of the G12A mutant full-length hammerhead ribozyme at pH 7.4. (A) is a plot of a subset of time points shown in (B). (C) is an independent experimental repeat of (A). At pH 7.4, the rate is approximately 0.0001/min in all three cases. A representative polyacrylamide gel is shown in the inset of (B). The bottom band is the accumulating product at various time points, and the top band is the reactant. At pH 8.4 (D), the rate is 10-fold faster, consistent with the log-linear relation between rate and pH observed in the chemical step of hammerhead reactions. The estimated rate (*) of the wild-type G12 hammerhead at pH 7.4 (extrapolated from results obtained at pH 6.5, due to the fast cleavage rate) is approximately 50/min. Hence the relative mutant to wild-type rate at pH 7.4 is approximately 0.000002, which is consistent with a 10^−6^-fold effect estimated using the differences in pK_a_ for G12 and A12 (i.e., pK_a_ = 9.5 − 3.5 = 6). Time-course assays were performed following the procedure described in Martick and Scott (2006) [13]. Briefly, 2 μl of ^32^P-γ-ATP-labeled hammerhead substrate (10 pmol/μl) and 3 μl of 100 μM hammerhead enzyme strand were combined with 2 μl of 1 M Tris-HCl (pH 7.4 or 8.4), 0.8 μl of 5 M NaCl, 1.8 μl of 2.25 mM EDTA, and 15.4 μl of water and heated to 95 °C for 2 min, then 65 °C for 2 min, and then cooled to 20 °C. A 3-μl aliquot was removed and added to 57 μl of standard PAGE loading buffer/dye and flash frozen, followed by addition of 15 μl of 25 mM MgCl_2_ to initiate the cleavage reaction. Aliquots were subsequently removed from the reaction and quenched at 10, 20, and 30 min and 1, 2, 3, 4, 5, 6, 12, 24, 36, 48, 72, and 120 h at pH 7.4 (A and B) and up to 12 h (C). At pH 8.4, aliquots were removed at 0, 5, 10, 20, 30, 45, and 60 min and 2, 3, 4, 5, 6,7, 8, 9, and 10 h (D). In each case, the aliquot was mixed with PAGE loading buffer/dye containing a 10-fold molar excess of EDTA to quench the reaction and was flash frozen.
(149 KB PDF)

### Accession Numbers

Coordinates and amplitudes for the cleaved (2QUW) and uncleaved (2QUS) structures are available in the Protein Data Bank (http://www.rcsb.org).

## Abbreviations

Smα: *Schistosoma mansoni* alpha repetitive sequence
sTRSV: satellite tobacco ringspot virus
