# Supplementary figures and images for: Capturing Hammerhead Ribozyme Structures in Action by Modulating General Base Catalysis
Source: PLoS Biol. 2008 Sep 30;6(9):e234. doi: 10.1371/journal.pbio.0060234 (PMC2553840; doi:10.1371/journal.pbio.0060234)

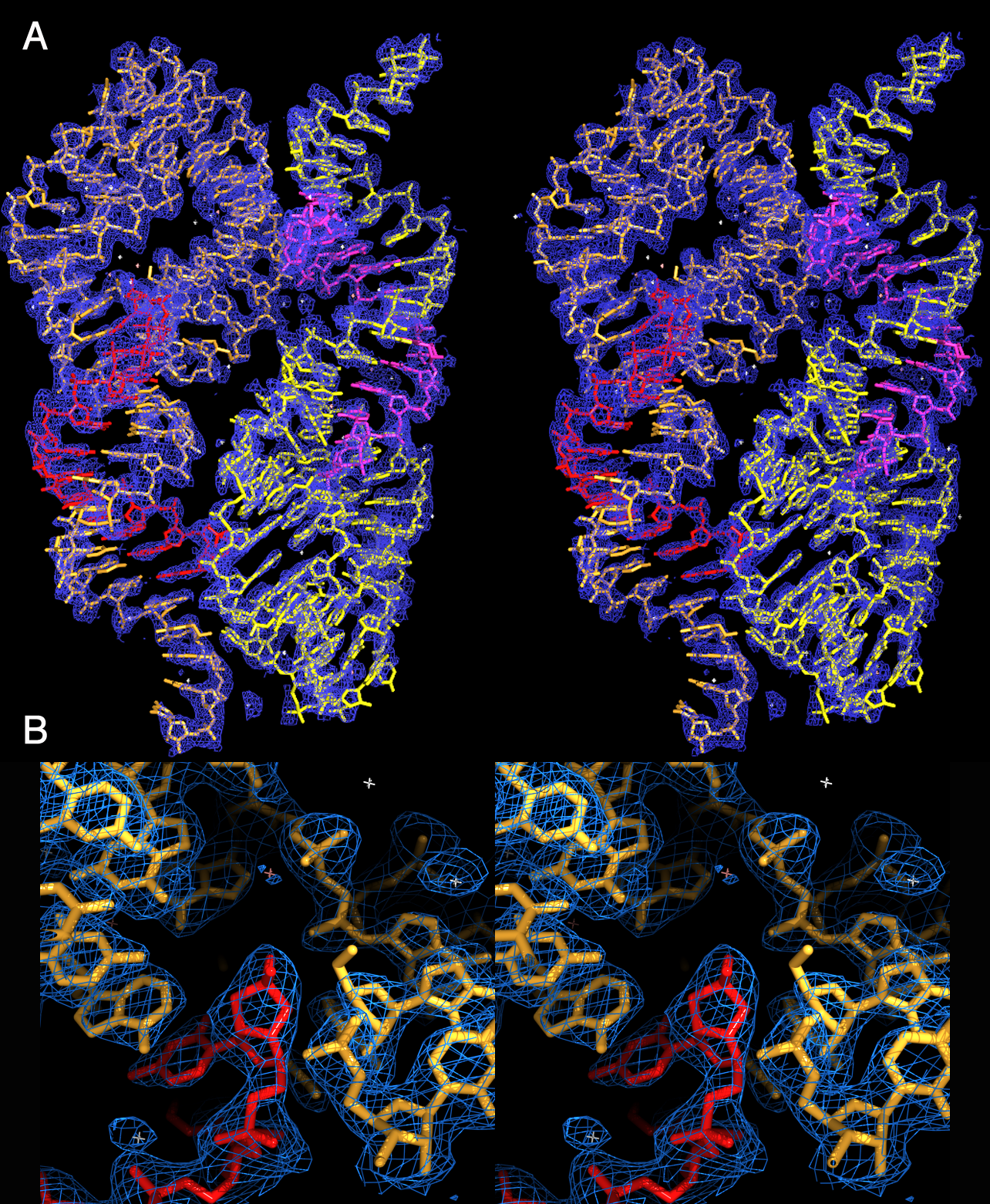

Supplement: Figure S1 — (A) Stereo view of a composite omit electron density map of the cleaved form of the hammerhead ribozyme at 2.2 Å resolution contoured at 1.0 root mean square deviation (RMSD). Each omit fragment in the composite was generated by omission of a unique 10% of the RNA structure, followed by simulated annealing refinement of the remainder of the structure (starting temperature 4,000 K) to reduce model phase bias, within the crystallographic refinement program CNS v. 1.2. [35]. (B) shows a close-up view of the active site of molecule 1. (6.59 MB TIF) [file pbio.0060234.sg001.tif]

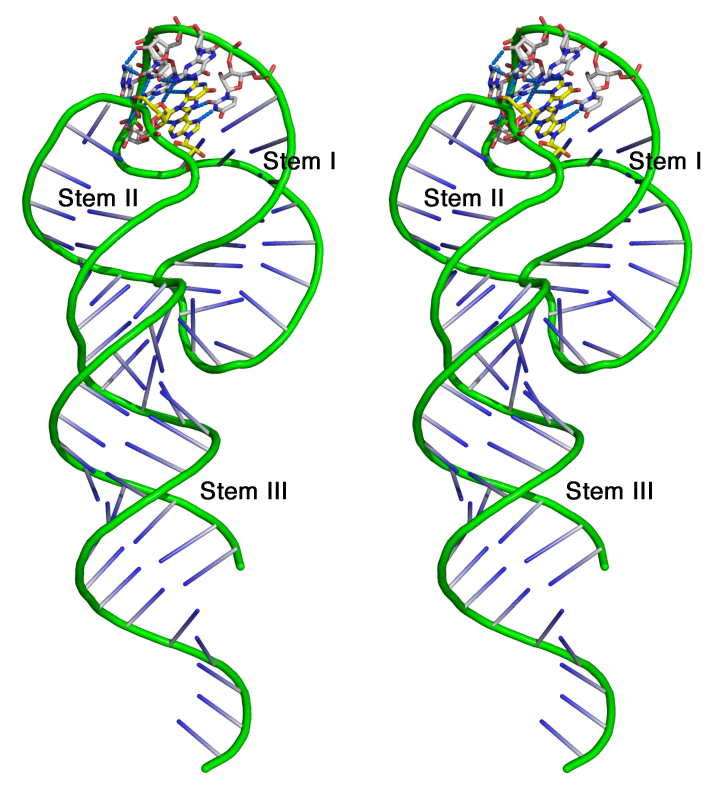

Supplement: Figure S2 — Overall stereo view of the sTRSV hammerhead backbone structure, with the nucleotides involved in the tertiary contacts shown explicitly. (752 KB TIF) [file pbio.0060234.sg002.tif]

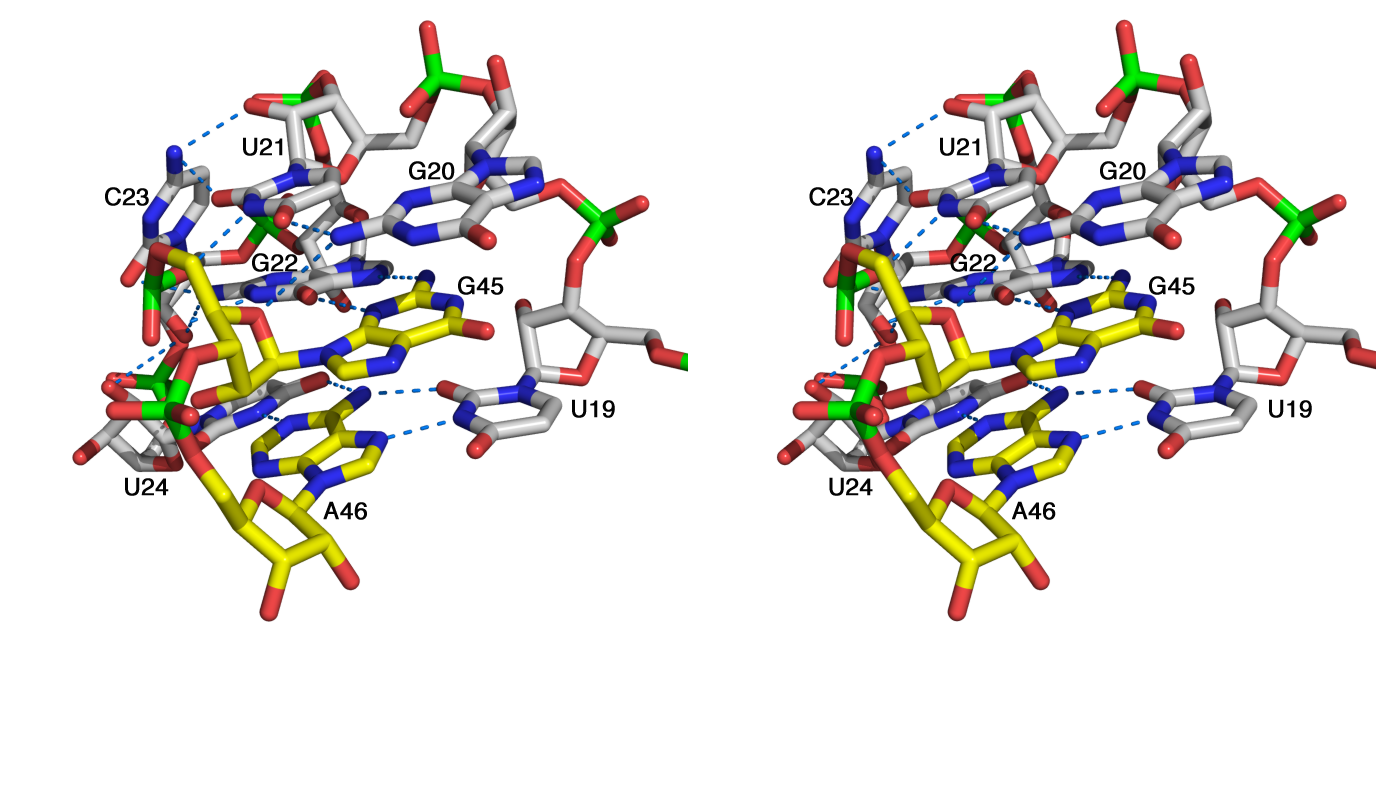

Supplement: Figure S3 — Close-up stereo view of the tertiary contacts shown in Figure S2, similar to Figure 5 (but without the backbone cartoon). (1.2 MB TIF) [file pbio.0060234.sg003.tif]
