# Supplementary material for: Capturing Hammerhead Ribozyme Structures in Action by Modulating General Base Catalysis
Source: PLoS Biol. 2008 Sep 30;6(9):e234. doi: 10.1371/journal.pbio.0060234 (PMC2553840; doi:10.1371/journal.pbio.0060234)
Supplement: Figure S4 — (A, B, and C) are the results of experiments that measure the rate of the G12A mutant full-length hammerhead ribozyme at pH 7.4. (A) is a plot of a subset of time points shown in (B). (C) is an independent experimental repeat of (A). At pH 7.4, the rate is approximately 0.0001/min in all three cases. A representative polyacrylamide gel is shown in the inset of (B). The bottom band is the accumulating product at various time points, and the top band is the reactant. At pH 8.4 (D), the rate is 10-fold faster, consistent with the log-linear relation between rate and pH observed in the chemical step of hammerhead reactions. The estimated rate (*) of the wild-type G12 hammerhead at pH 7.4 (extrapolated from results obtained at pH 6.5, due to the fast cleavage rate) is approximately 50/min. Hence the relative mutant to wild-type rate at pH 7.4 is approximately 0.000002, which is consistent with a 10−6-fold effect estimated using the differences in pKa for G12 and A12 (i.e., pKa = 9.5 − 3.5 = 6). Time-course assays were performed following the procedure described in Martick and Scott (2006) [13]. Briefly, 2 μl of 32P-γ-ATP-labeled hammerhead substrate (10 pmol/μl) and 3 μl of 100 μM hammerhead enzyme strand were combined with 2 μl of 1 M Tris-HCl (pH 7.4 or 8.4), 0.8 μl of 5 M NaCl, 1.8 μl of 2.25 mM EDTA, and 15.4 μl of water and heated to 95 °C for 2 min, then 65 °C for 2 min, and then cooled to 20 °C. A 3-μl aliquot was removed and added to 57 μl of standard PAGE loading buffer/dye and flash frozen, followed by addition of 15 μl of 25 mM MgCl2 to initiate the cleavage reaction. Aliquots were subsequently removed from the reaction and quenched at 10, 20, and 30 min and 1, 2, 3, 4, 5, 6, 12, 24, 36, 48, 72, and 120 h at pH 7.4 (A and B) and up to 12 h (C). At pH 8.4, aliquots were removed at 0, 5, 10, 20, 30, 45, and 60 min and 2, 3, 4, 5, 6,7, 8, 9, and 10 h (D). In each case, the aliquot was mixed with PAGE loading buffer/dye containing a 10-fold molar excess of EDTA to [file pbio.0060234.sg004.pdf]

Kinetic analysis of the G12A mutation

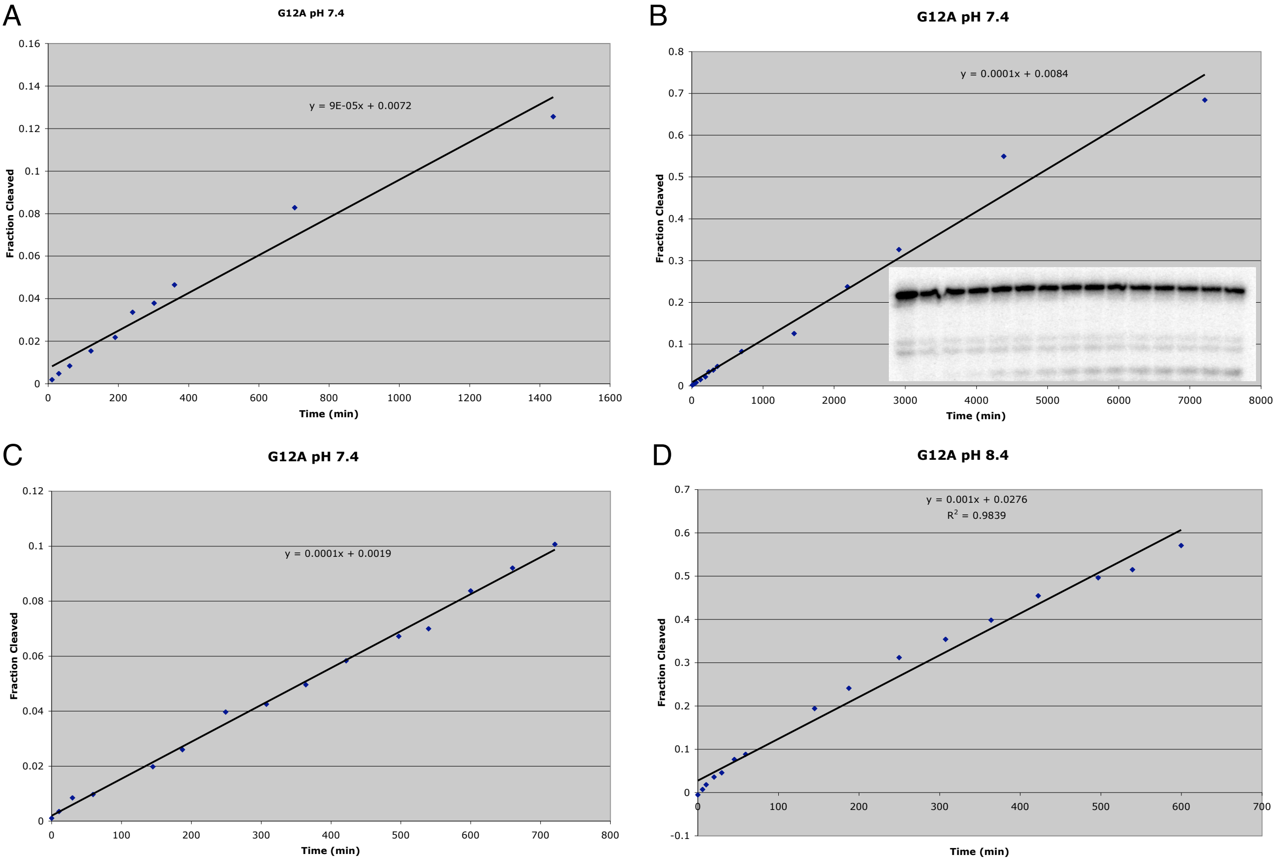

Condition	Rate	Fraction	Est (ΔpKa)
G12 (wild type) pH 7.4*	~50/min	1	1
G12A pH 7.4	~ 0.0001/min	~0.000002	0.000001
G12A pH 8.4	~ 0.001/min		
